# Establishment of the Normative Value of Classical Bluestone’s Nine-Step Inflation/Deflation Tympanometric Eustachian Tube Function Test

**DOI:** 10.3390/diagnostics14242810

**Published:** 2024-12-13

**Authors:** Jing-Jie Wang, Rong-San Jiang, Chien-Hsiang Weng

**Affiliations:** 1 Department of Otolaryngology, Taichung Veterans General Hospital, Taichung 40705, Taiwan; nathan07302003@vghtc.gov.tw; 2 School of Medicine, National Yang Ming Chiao Tung University, Taipei 112304, Taiwan; 3 Department of Medical Research, Taichung Veterans General Hospital, Taichung 40705, Taiwan; 4 Department of Otolaryngology, Tungs’ Taichung Metro Harbor Hospital, Taichung 435403, Taiwan; 5 Department of Family Medicine, Warren Alpert Medical School, Brown University, Providence, RI 02912, USA; 6 Brown Health Medical Group Primary Care, Brown University Health, Providence, RI 02903, USA

**Keywords:** Eustachian tube function, Eustachian tube dysfunction, nine-step inflation–deflation tympanometric Eustachian tube function test, maximal peak pressure difference, seven-item Eustachian Tube Dysfunction Questionnaire

## Abstract

Background/Objectives: The nine-step inflation/deflation tympanometric Eustachian tube function test (commonly referred to as the nine-step test) is a widely utilized method for evaluating Eustachian tube function (ETF). This study aimed to establish normative values for the nine-step test to facilitate the diagnosis of Eustachian tube dysfunction (ETD). Methods: A total of 160 adults, including 70 healthy volunteers and 90 patients with chronic rhinosinusitis (CRS), were recruited for this study. Participants were further categorized into “fair ETF” and “poor ETF” groups based on their scores on the Eustachian Tube Dysfunction Questionnaire (ETDQ-7). Eustachian tube function was assessed using both the nine-step test and the ETDQ-7. The diagnostic accuracy of the maximal peak pressure difference (MPD) from the nine-step test was evaluated, using an ETDQ-7 score of ≥14 as the reference standard. Discriminative ability was analyzed using receiver operating characteristic (ROC) curves. Results: An MPD value of ≤4 yielded an area under the ROC curve (AUC) of 0.619, indicating moderate discriminative ability in the Taiwanese population. The median MPD value on the nine-step test was 9.5 (interquartile range [IQR]: 4.5–14.0) in participants with an ETDQ-7 score of <14, compared to a median MPD value of 7.5 (IQR: 2.5–12.0) in those with an ETDQ-7 score of ≥14 (*p* = 0.033). This finding suggests a potential association between MPD values and ETDQ-7 scores. Conclusions: This study identified an MPD value of 4 as a normative cutoff for screening ETD in a Taiwanese population. However, the diagnostic discriminative power of this parameter was moderate.

## 1. Introduction

The Eustachian tube (ET) is a dynamic tubular structure connecting the middle ear cavity to the nasopharynx, serving as the sole pathway for ventilation and pressure equalization within the middle ear. It also facilitates mucociliary clearance, prevents the reflux of sound and fluid from the nasopharynx, and protects against sniff-induced high negative middle ear pressure. The primary function of the ET is to regulate air pressure in the middle ear, thereby ensuring efficient sound transmission. Clearance of middle ear secretions occurs via a combination of muscular peristaltic action within the Eustachian tube and the mucociliary escalator. Any dysfunction of the ET can lead to Eustachian tube dysfunction (ETD), which may manifest as negative middle ear pressure, aural fullness, muffled hearing, hearing loss, popping sounds, tinnitus, otalgia, intermittent sharp ear pain, disequilibrium, and autophony [1,2,3,4,5,6,7].

The prevalence of ETD has been reported to range widely, from 1% to 70%, depending on the population studied and diagnostic criteria used [3,6,8,9,10]. In the United States, the prevalence of ETD is estimated to be approximately 6.1% among children [11], 4.4% among adolescents [12], 4.6% among adults [5], 5.44% among the elderly, and 9.08% in cancer patients [13]. In Europe, ETD affects over 1% of the adult population, while approximately 40% of children experience at least temporary episodes of ETD [8,9]. Additionally, studies indicate that nearly 70% of patients undergoing tympanoplasty for middle ear disorders develop ETD [6,10]. ETD is a prevalent but poorly understood condition, recognized as a significant etiological factor contributing to symptoms such as aural fullness and increased listening effort [1]. These findings highlight the critical need for increased awareness, focused clinical attention, and further research to better understand and manage this condition.

Eustachian tube dysfunction is defined by symptoms and clinical signs of pressure dysregulation in the middle ear, as outlined in the international consensus guidelines [2]. It is characterized by the Eustachian tube’s inability to maintain adequate pressure equalization or mucociliary transport and can be broadly classified into three categories: dilatory ETD (further subdivided into functional obstruction, dynamic dysfunction due to muscular failure, and anatomical obstruction), baro-challenge-induced ETD, and patulous ETD [2,14]. Notably, patients may experience fluctuations along this disease spectrum, transitioning between different subtypes of the condition.

The diagnosis of ETD is based on a combination of clinical history, physical examination, tympanometry, audiometry, and other diagnostic tests. A recent clinical consensus statement specifically defined dilatory ETD based on patient history and/or evidence of negative middle ear pressure [15]. Both subjective assessments and objective evaluations are critical for the differential diagnosis of ETD subtypes. However, standardized diagnostic protocols remain lacking in this field, underscoring the need for further research and consensus development [1,2,16,17].

From the subjective assessment perspective, McCoul et al. developed and performed an initial validation of the seven-item Eustachian Tube Dysfunction Questionnaire (ETDQ-7), a disease-specific instrument designed to evaluate symptoms associated with ETD. The study demonstrated that the ETDQ-7 exhibits acceptable internal consistency and strong test–retest reliability, establishing it as a valid and reliable tool for assessing symptoms in adult patients with ETD [17].

Although various objective tests, such as sonotubometry and tubo-tympano-aerodynamic-graphy (TTAG), have been developed to evaluate Eustachian tube function and are commonly employed in routine clinical practice, each method has distinct limitations [1]. A review of the literature indicated that the nine-step inflation-deflation tympanometric Eustachian tube function test (commonly referred to as the nine-step test), originally developed by Bluestone in 1975, demonstrates moderate diagnostic accuracy for both dilatory ETD (area under the curve [AUC] = 0.875) and patulous ETD (AUC = 0.769), as reported by Bae et al. [18]. The nine-step test is also one of the most commonly utilized tools for evaluating Eustachian tube function in major medical centers [1]. Accordingly, we selected this objective test as the focus of our study, with the aim of generating findings that could enhance clinicians’ confidence in diagnosing ETD.

In light of the absence of standardized diagnostic protocols in this field, this study aims to advance the understanding of the most commonly utilized subjective and objective tools for evaluating Eustachian tube function (ETF) to improve diagnostic accuracy and inform clinical management. Furthermore, this study seeks to establish normative values for the nine-step test by comparing its outcomes with those of the widely used ETDQ-7.

## 2. Methods

### 2.1. Study Population

This study involved secondary analyses conducted on a prospective cohort of 160 adult participants (aged ≥ 18 years) between January 2020 and January 2024. Of these, 70 individuals were healthy volunteers who underwent rigorous screening by a board-certified otolaryngologist to exclude the presence of nasal or ear diseases. Exclusion criteria for this group included a history of nasal allergies, recent upper respiratory infections, immunodeficiency, pregnancy, or the use of oral or nasal corticosteroids (e.g., prednisolone) or antihistamines, to minimize potential confounding factors that could affect nasal or Eustachian tube function.

The remaining 90 participants were patients diagnosed with chronic rhinosinusitis (CRS), recruited over a four-year period from January 2020 to January 2024. These CRS patients were selected from a cohort scheduled for functional endoscopic sinus surgery, representing a population with well-documented sinonasal pathology, potentially affecting Eustachian tube function. To ensure cohort homogeneity, patients with a history of middle ear disease, which could independently influence the outcomes of interest, were excluded. The inclusion of CRS patients was based on prior evidence highlighting the high prevalence of ETD in this population, underscoring the relevance of investigating this subgroup [19,20].

All enrolled participants completed the ETDQ-7 to provide a subjective assessment of ETD symptoms, a tool validated in previous studies for its sensitivity and specificity in detecting Eustachian tube-related symptoms. Following the questionnaire, each participant underwent a standardized nine-step inflation/deflation tympanometric Eustachian tube function test, performed in a controlled clinical setting. This sequential approach allowed for a comprehensive evaluation of both subjective and objective measures of Eustachian tube function across the study cohort, facilitating a comparative analysis between the fair and poor ETF groups.

In this study, the cohort was divided into two groups based on ETDQ-7 scores, a subjective measure of Eustachian tube function (ETF). Participants with an ETDQ-7 score below 14 were categorized as having fair ETF, while those with a score of 14 or higher were classified as having poor ETF. These groupings facilitated subgroup analyses to examine differences in ETF between healthy individuals and those affected by CRS.

### 2.2. Eustachian Tube Dysfunction Questionnaire 7 (ETDQ-7)

The ETDQ-7 is a validated, symptom-specific tool used to assess the presence and severity of ETD. This questionnaire comprises seven items addressing common ETD symptoms: (1) a sensation of pressure in the ears; (2) ear pain; (3) a feeling of fullness or the sensation that ears are clogged or submerged; (4) ear symptoms exacerbated by upper respiratory conditions, such as colds or sinusitis; (5) auditory sensations of crackling or popping; (6) tinnitus, or ringing in the ears; and (7) the perception of muffled hearing. Each item is scored on a Likert scale from 1 to 7, where 1 indicates no problem and 7 indicates the most severe degree of symptom burden over the previous month.

The cumulative ETDQ-7 score ranges from 7 to 49, with higher scores indicating more severe symptoms and a greater impact on quality of life. In studies utilizing the English version of the ETDQ-7, a receiver operating characteristic (ROC) cutoff score of ≥14.5 has been identified as indicative of ETD [17]. This threshold has been validated across diverse patient populations, establishing it as a reliable diagnostic standard for ETD. It is widely employed in both research and clinical settings to quantify symptom severity and guide subsequent intervention decisions.

The English version of the ETDQ-7 was translated into Mandarin by Lin et al., with validation performed by the same research group, achieving 100% sensitivity and specificity [21]. To minimize cultural and linguistic confounding factors, we adopted this Mandarin version of the ETDQ-7 as the gold standard tool for assessing ETD in our study. In Lin et al.‘s study using the Mandarin version, the ROC cutoff score for diagnosing ETD was determined to be ≥13.5. Based on this, we defined the ETD group in our study as participants with an ETDQ-7 score ≥14.

### 2.3. Nine-Step Inflation/Deflation Tympanometric Eustachian Tube Function Test

The objective ETF was evaluated by using GSI TympStar Pro (Grason-Stadler Inc., Eden Prairie, MN, USA) for the nine-step inflation–deflation tympanometric test. One limitation to note is that the device (GSI TympStar Pro) used in this study was a modified version of the nine-step test originally conceptualized by Bluestone in 1975 [18,22].

The nine-step inflation–deflation tympanometric test was performed according to the following: (1) Resting middle ear pressure was recorded via tympanometry. (2) Ear canal pressure was increased to +200 mmH_2_O, causing medial deflection of the tympanic membrane and an increase in middle ear pressure. The subject swallowed to equilibrate the resulting middle ear overpressure. (3) Without swallowing, ear canal pressure was normalized, creating a slight negative middle ear pressure as the tympanic membrane moved outward, which was documented by the tympanogram (post-inflation). (4) The subject then attempted to equilibrate the negative middle ear pressure by swallowing, allowing airflow from the nasopharynx to the middle ear if equilibration was achieved. (5) The tympanogram recorded the degree of equilibration. (6) Ear canal pressure was decreased to −200 mmH_2_O, producing lateral deflection of the tympanic membrane and a corresponding reduction in middle ear pressure. The subject swallowed to equilibrate the negative middle ear pressure, facilitating airflow from the nasopharynx to the middle ear. (7) Without swallowing, ear canal pressure was normalized, establishing a slight positive pressure in the middle ear as the tympanic membrane moved medially, which was recorded as post-deflation overpressure (post-deflation). (8) The subject swallowed to reduce the overpressure, permitting airflow from the middle ear to the nasopharynx if equilibration occurred. (9) The final tympanogram documented the extent of this equilibration. A “shift” value between the peaks of tympanometry from steps 1/3/7, named MPD, was then calculated, representing the functional test outcome. The MPD value is commonly used for ETF evaluation. Higher values of MPD indicate potentially better Eustachian tube function [22]. That is, the participants in our study who did not pass either the post-inflation (step 3) or post-deflation (step 7) portions of the test were classified as having poor nine-step test results, while those who passed both were considered to have good results. According to Fernau et al. and McBride et al., an MPD value of 10 daPa can be considered a cutoff point in the nine-step test [23,24].

### 2.4. Analysis Protocol Design

This study aimed to evaluate the clinical utility and discriminative capability of the nine-step inflation/deflation tympanometric test in diagnosing ETD among Taiwanese adults by conducting a comparative analysis with the established ETDQ-7. The ETDQ-7 scores were used as a reference standard for ETF, enabling the classification of the 160 adult participants into two distinct groups: those with scores of 14 or higher, indicating fair ETF, and those with scores below 14, indicating poor ETF. Based on this categorization, 126 participants were classified as having fair ETF, while 34 were identified as having poor ETF, thereby facilitating subgroup analyses of the diagnostic efficacy of the nine-step test in relation to self-reported ETD symptoms.

This research involved a secondary analysis of data from a prospective cohort of 160 adults, comprising 70 healthy individuals and 90 patients. The data derived from the healthy participants were pivotal for calculating the area under the receiver operating characteristic curve (AUC) for both the ETDQ-7 and the nine-step tympanometric test, providing a robust basis for evaluating their diagnostic performance.

This study was designed to validate the applicability of the nine-step tympanometric test in clinical settings and to assess its ability to differentiate varying levels of Eustachian tube function within a Taiwanese cohort. By correlating objective findings from the tympanometric test with subjective symptom severity as measured by the ETDQ-7, this study sought to establish the nine-step test as a reliable diagnostic adjunct for ETD evaluation.

Ethical approval for the study protocol was obtained from the Institutional Review Board of Taichung Veterans General Hospital (IRB No. CE23522C), ensuring compliance with ethical standards for research involving human subjects. Informed consent was secured in writing from all participants following a detailed explanation of the study’s objectives, procedures, and potential risks. This rigorous ethical oversight underscores this study’s commitment to participant safety, privacy, and autonomy.

### 2.5. Statistical Analysis

We presented descriptive statistics for the study variables, using counts and percentages to summarize binary or categorical variables, while continuous variables were reported as medians with interquartile ranges (IQRs) to capture the distribution’s central tendency and variability. Statistical comparisons between groups were conducted using the Chi-square (Χ^2^) test for categorical variables and the nonparametric Mann–Whitney U test for continuous variables, given the non-normal distribution of some data.

To assess the diagnostic accuracy and discriminative capability of each test, we calculated the AUC for the ETDQ-7 and the ETF test. A receiver operating characteristic (ROC) curve was generated for each test by plotting the sensitivity (true-positive rate) against the false-positive rate (1-specificity) across a range of threshold values. This curve illustrates the balance between sensitivity and specificity as the decision threshold changes, providing insight into each test’s diagnostic performance. The AUC, computed as the integral under the ROC curve, serves as a comprehensive measure of the test’s classification ability, with values ranging from 0.5 (no discriminative ability) to 1.0 (perfect classification). Higher AUC values indicate superior discriminative power, with values closer to 1.0 reflecting strong accuracy in distinguishing between cases with and without ETD.

All statistical analyses were two-tailed, with *p* < 0.05 considered indicative of statistical significance. The results are reported with 95% confidence intervals (95% CIs) to ensure precision in effect estimates, and *p*-values are provided to denote the strengths of associations. All analyses were performed using STATA software, version 13.1 (StataCorp, College Station, TX, USA), which facilitated both descriptive and inferential statistical procedures.

## 3. Results

In the fair ETF group (ETDQ-7 < 14), the mean age was 37.0 years (interquartile range [IQR] 30.0–51.0), whereas participants in the poor ETF group (ETDQ-7 ≥ 14) had a significantly higher mean age of 51.5 years (IQR 43.0–60.0), with a *p*-value of <0.001 indicating a statistically significant age difference between the two groups. A gender disparity was also observed, with 77% of participants in the fair ETF group identifying as female, compared to only 14% in the poor ETF group, a statistically significant difference with *p* = 0.037. These findings suggest potential age- and sex-related differences in the prevalence of ETF, warranting further investigation into demographic influences on Eustachian tube health (Table 1).

An objective assessment of ETF was conducted using the nine-step inflation/deflation tympanometric test, with the MPD serving as the primary quantitative indicator. The median MPD value for participants in the fair ETF group was 9.5 (IQR 4.5–14.0), while those in the poor ETF group had a lower median MPD of 7.5 (IQR 2.5–12.0). This difference in MPD values between the two groups was statistically significant (*p* = 0.033), suggesting a possible inverse relationship between MPD values on the nine-step test and the severity of symptoms as reported on the ETDQ-7. The lower MPD observed in the poor ETF group may reflect reduced Eustachian tube function, aligning with self-reported ETD severity as measured by the ETDQ-7 (Table 1).

Table 2 presents a comparative analysis of ETDQ-7 scores between individuals with and without CRS. The participants were categorized into two groups, those with CRS (n = 90) and those without CRS (n = 70), comprising a total sample size of 160. The median ETDQ-7 score for the overall cohort was 9 (interquartile range [IQR]: 7–12). Participants diagnosed with CRS exhibited a higher median ETDQ-7 score of 11 (IQR: 7–16.25) compared to a median score of 7 (IQR: 7–9) among those without CRS. This difference was statistically significant, with a *p*-value of <0.001. In terms of participants with ETDQ-7 scores ≥14, 34 individuals (21.25% of the total cohort) met this threshold. Notably, all participants with scores ≥14 were within the CRS group, representing 37.78% of this subgroup, whereas none of the participants in the non-CRS group reached this threshold. This comparison also yielded a highly statistically significant *p*-value (<0.001). These findings highlight a strong and statistically significant association between CRS and elevated ETDQ-7 scores, underscoring the relevance of ETDQ-7 as a potential indicator of disease burden in CRS populations.

The correlations between the ETDQ-7 total scores and the nine-step test MPD values are summarized in Table 3. The correlation between the ETDQ-7 total score and the nine-step test MPD value was weakly negative and not statistically significant (*p* = 0.217). Age demonstrated a significant positive correlation with the ETDQ-7 total score (*p* < 0.001) and a significant negative correlation with the nine-step test MPD value (*p* = 0.007). These findings suggest that while age is significantly associated with both ETDQ-7 total scores and MPD values, no significant relationship exists between the ETDQ-7 scores and the nine-step test MPD values.

**Figure 1:**
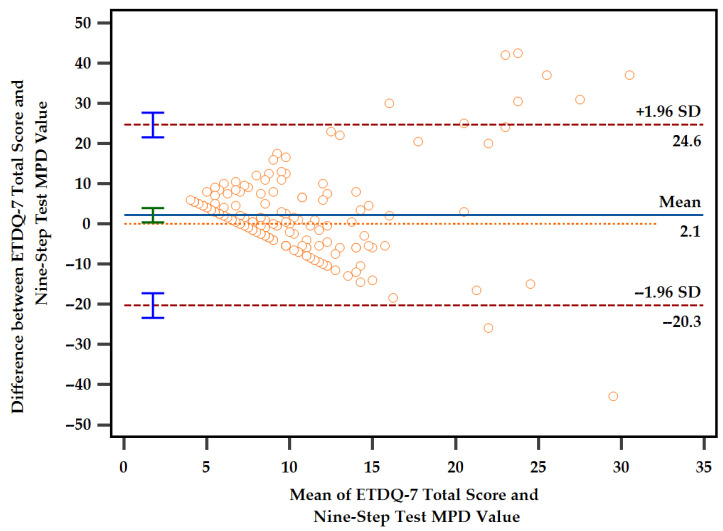
Comparison of the ETDQ − 7 total score and the nine-step test MPD value. ETDQ − 7: seven-item Eustachian Tube Dysfunction Questionnaire; Nine-step test: nine-step inflation–deflation tympanometric Eustachian tube function test; MPD: maximal peak pressure difference.

**Figure 2:**
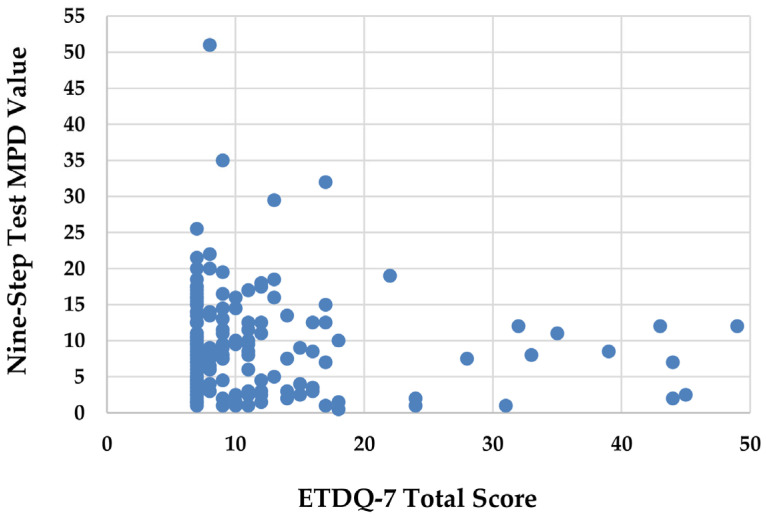
Variation in MPD value according to ETDQ-7 total score. ETDQ-7: seven-item Eustachian Tube Dysfunction Questionnaire. Nine-step test: nine-step inflation–deflation tympanometric Eustachian tube function test. MPD: maximal peak pressure difference.

A Bland–Altman plot was employed to evaluate the consistency between the ETDQ-7 total scores and the nine-step test MPD values in assessing ETF. As shown in Figure 1, no correlation was observed between the two measures (rs = –0.098). Furthermore, the Bland–Altman plot revealed a mean difference of 2.13 (95% CI: 0.34–3.93; *p* = 0.020), indicating that the assessment results of the two methods are inconsistent. These findings suggest that the ETDQ-7 and the nine-step test are not interchangeable in evaluating ETF. Specifically, there was considerable variability in MPD values across a range of ETDQ-7 scores, especially among those with an ETDQ-7 score <20. This wide distribution of MPD values in participants with lower ETDQ-7 scores (representing fewer symptoms) suggests that the nine-step test may be particularly variable among individuals with mild or moderate ETF symptoms, as depicted in Figure 2.

**Figure 3:**
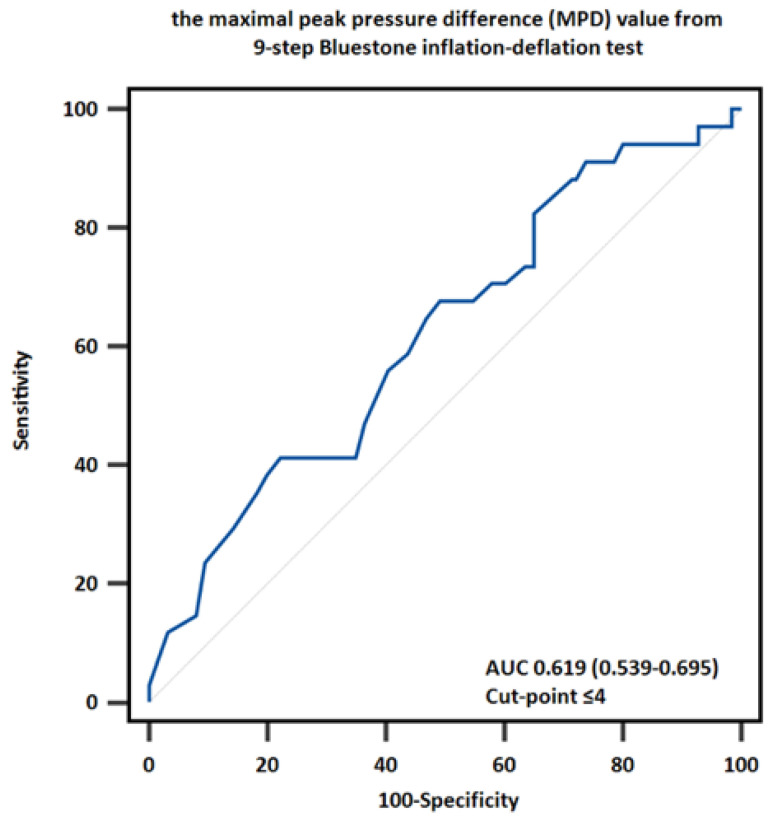
The discriminative ability of the MPD value in identifying Eustachian tube dysfunction.

For diagnostic purposes, an ETDQ-7 score of ≥14 was established as the gold standard for identifying clinically significant ETD. Analysis of MPD cutoffs indicated that a threshold of ≤4 provided the highest diagnostic accuracy, with an AUC of 0.619 (95% CI: 0.539–0.695). This finding suggests that when the MPD value exceeds 4, there is a 77.78% likelihood that the ETDQ-7 score will be <14, indicating the absence of clinically significant ETD. However, the AUC value of 0.619, being below the commonly accepted threshold of 0.7, reflects only moderate discriminative performance. This outcome highlights the limited accuracy of the nine-step tympanometric test in distinguishing between fair and poor ETF when compared to the subjective ETDQ-7 measure (Figure 3). The sensitivity and specificity of the MPD cutoff were 41.18% and 77.78%, respectively.

**Figure 4:**
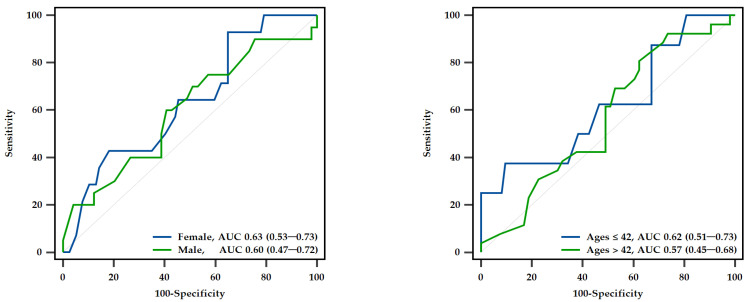
The discriminative capability of the MPD value in diagnosing Eustachian tube dysfunction, stratified by gender and age. MPD: maximal peak pressure difference; AUC: area under the receiver operating characteristic curve.

Given the significant differences in age and gender between the fair and poor ETF groups based on the ETDQ-7 total scores, a subgroup ROC analysis was conducted based on gender and age. The comparison of ROC curves between males and females indicated no significant difference (*p* = 0.770). The AUC for females was 0.632 (95% CI: 0.525–0.731), and the AUC for males was 0.599 (95% CI: 0.474–0.716), with neither achieving statistical significance. Similarly, the analysis stratified by the median age (42 years) showed no significant differences between the ROC curves for the two age groups (*p* = 0.682). The AUC for individuals aged 18–42 years was 0.621 (95% CI: 0.506–0.726), whereas the AUC for those over 42 years was 0.565 (95% CI: 0.449–0.677), with neither group reaching statistical significance (Figure 4).

## 4. Discussion

The Eustachian tube plays a critical role in middle ear ventilation, pressure equalization, and mucociliary clearance, while also preventing the reflux of sound and fluids from the nasopharynx and mitigating the development of excessive negative middle ear pressure induced by sniffing. Any deficiency in this tube can lead to ETD. The causes of ETD are highly diverse, with common associations found in inflammatory diseases, such as rhinitis or CRS [5,14]. Baro-challenge-induced ETD refers to the failure of Eustachian tube opening in specific situations, such as during deep-sea diving or altitude descent, when atmospheric pressure increases. In contrast, patulous ETD occurs when the Eustachian tube remains abnormally open, causing continuous communication between the nasopharynx and middle ear. This condition often leads to autophony, where patients perceive their own voice as excessively loud, prompting habitual sniffing to alleviate the sensation. Patients may fluctuate along the spectrum of ETD, even between subtypes, highlighting the difficulty and importance of differential diagnosis [1,2,3,4,5,6,7].

To date, no definitive “gold standard” objective test for diagnosing ETD has been established. However, expert consensus recommends that ETD diagnosis should be based on a combination of clinical history, physical examination, tympanometry, audiometry, and additional tests as clinically indicated [15]. The absence of established standard diagnostic protocols in this field is noted [1,2,16,17].

Although several objective tests for assessing Eustachian tube function have been developed, and some are employed in routine clinical practice, each test presents significant limitations. The available data on diagnostic accuracy remain limited and exhibit variability in quality, largely due to inconsistencies in comparative testing methodologies and the diverse spectrum of otological disorders associated with ETD [1]. A review of the literature indicated that the classical nine-step inflation–deflation Eustachian tube function test, a method derived from the inflation–deflation testing first developed by Bluestone in 1975, demonstrates moderate diagnostic accuracy for both obstructive ETD (area under the curve [AUC] = 0.875) and patulous ETD (AUC = 0.769), as reported by Bae et al. [18]. The nine-step test alone can identify 81% of healthy ET; furthermore, this test combined with sonotubometry can identify 96% of healthy ET [25]. In addition, the test could detect ET opening in 81% of healthy ears according to McBride et al. [25].

Our study identified an MPD value of 4 as a normative cutoff for screening ETD in the Taiwanese population, though its discriminative power was moderate. This finding indicates that the MPD value derived from the nine-step tympanometric test is not a reliable diagnostic marker for ETD when using an ETDQ-7 score of ≥14 as the reference standard, despite the widespread use of the nine-step test globally. The highest observed AUC was 0.619 for an MPD threshold of ≤4, signifying only moderate discriminative ability in detecting ETD. Specifically, when the MPD value exceeded 4, there was a 77.78% likelihood that the ETDQ-7 score was <14, suggesting the absence of ETD. Conversely, the likelihood of confirming ETD when the MPD value was ≤4 was only 41.18%. Furthermore, the AUC of 0.619 reported in our study is lower than that in other studies, reflecting the moderate discriminative capacity of MPD values in our study population. The sensitivity and specificity of the MPD cutoff were 41.18% and 77.78%, respectively, which are notably lower than the sensitivity and specificity reported for the ETDQ-7 cutoff. According to McCoul et al. (2012) [17], the ETDQ-7 score demonstrated both 100% sensitivity and specificity. Importantly, no correlation was observed between the ETDQ-7 and the nine-step test, highlighting that these two measures are not interchangeable for evaluating ETF.

Our findings in this study underscore the partial but limited utility of the nine-step tympanometric test as a standalone objective measure of ETF, particularly in detecting clinically relevant ETD as defined by the ETDQ-7. The observed variations in ETF based on age and sex indicate that demographic factors may play a role in influencing ETF. However, the subgroup ROC analysis stratified by gender and age did not reveal statistical significance in either group, underscoring the necessity for further research to clarify these associations. Importantly, our analysis identified a significant relationship between age and both ETDQ-7 total scores and MPD values. Conversely, no significant correlation was found between ETDQ-7 scores and MPD values obtained from the nine-step test. In clinical practice, the integration of objective assessments, such as the nine-step test, with subjective evaluation tools like the ETDQ-7, could provide a more comprehensive evaluation of ETF, particularly in populations with varying symptom severities. These findings highlight the intricate nature of ETD diagnosis and underscore the potential advantages of employing integrated diagnostic methodologies to improve patient assessment and management.

Despite the nine-step test exhibiting a limited discriminative power, there remained a significant difference in MPD values between the fair ETF group (ETDQ-7 < 14) and the poor ETF group (ETDQ-7 ≥ 14), with median values of 9.5 and 7.5, respectively. This finding suggested a potential relationship between the ETD, low MPD value, and high ETDQ-7 scores.

Notably, the MPD, a parameter indicative of normal ETF in the nine-step test, varies across studies. The MPD has been reported to range from 10 to 15, with variations potentially attributable to differences among racial populations [18,23,26,27,28]. The reference MPD cutoff value initially suggested by the tympanometry manufacturer is 15; however, this has not been validated. In the early literature, the Handbook of Clinical Impedance Audiometry, Bluestone et al. stated that a change of at least 10 daPa in middle ear pressure during swallowing in the post-inflation and post-deflation phases of the test suggested the presence of ETD. Afterward, McBride et al. and Fernau et al. followed this reference value for their studies [23,25]. A recent study found an MPD value of 13 could have been a proper cutoff value in a Korean population [18]. In our study, an MPD value of ≤4 yielded the highest AUC of 0.619, which indicated that an MPD value of 4 could have been a normative cutoff value in the Taiwanese population.

This secondary analysis of a prospective cohort study has several limitations. First, the relatively small sample size may have limited the statistical power of the findings. Second, as all study participants were of Taiwanese descent, the generalizability of the results to other racial and ethnic groups may be constrained. Additionally, the participant pool primarily consisted of young females, which may have contributed to the presence of outliers in the scatter plots for both the ETDQ-7 and the nine-step test. Lastly, the device used in this study (GSI TympStar Pro) represents a modified version of the nine-step test initially conceptualized by Bluestone in 1975. This modification may account for differences in the reference normative MPD values compared to those reported in other studies.

## 5. Conclusions

Given the lack of standardized diagnostic protocols in this field, this study focused on the most commonly employed subjective and objective tools for evaluating ETF. Among these, the nine-step test is one of the most widely used objective methods for ETF evaluation in major medical centers, making it the focal point of our investigation. However, our findings revealed that the MPD value derived from the nine-step test is not a reliable diagnostic marker for ETD when using an ETDQ-7 score of ≥14 as the reference standard. This suggests that combining the nine-step test with other objective methods, such as sonotubometry or TTAG, alongside subjective assessments like the ETDQ-7, could provide a more comprehensive and confirmatory approach to diagnosing ETD. Furthermore, our results indicated that an MPD value of 4 in the nine-step test served as a normative cutoff for screening ETD in the Taiwanese population, although the test demonstrated only moderate discriminative power.

## Figures and Tables

**Table 1:** Comparison of demographic characteristics between fair and poor ETF groups.

	Total (n = 160)		ETDQ-7 Total Score				*p*-Value
			<14 (n = 126) Fair ETF		≥14 (n = 34) Poor ETF		
Age, median (IQR)	42	(31.25–53)	37	(30–51)	51.5	(42.75–60.75)	<0.001 **
Age, n (%)							0.023 *
18–29	32	(20.00%)	30	(23.81%)	2	(5.88%)	
30–64	116	(72.50%)	89	(70.63%)	27	(79.41%)	
≥65	12	(7.50%)	7	(5.56%)	5	(14.71%)	
Sex, n (%)							0.037 *
Female	91	(56.88%)	77	(61.11%)	14	(41.18%)	
Male	69	(43.13%)	49	(38.89%)	20	(58.82%)	
Nine-step test MPD value, median (IQR)	8.5	(4–12.88)	9.5	(4.5–14)	7.5	(2.38–12)	0.033 *

Mann–Whitney U test. Chi-square test. Fisher’s exact test. * *p* < 0.05, ** *p* < 0.01.

**Table 2:** Comparison of demographic characteristics between with and without CRS groups.

	Total (n = 160)		Enrollment				*p*-Value
			CRS (n = 90)		Without CRS (n = 70)		
Age, median (IQR)	42	(31.25–53)	51	(39–59)	33	(28–41.25)	<0.001 **
Age, n (%)							<0.001 **
18–29	32	(20.00%)	9	(10.00%)	23	(32.86%)	
30–64	116	(72.50%)	69	(76.67%)	47	(67.14%)	
≥65	12	(7.50%)	12	(13.33%)	0	(0.00%)	
Sex, n (%)							<0.001 **
Female	91	(56.88%)	35	(38.89%)	56	(80.00%)	
Male	69	(43.13%)	55	(61.11%)	14	(20.00%)	
ETDQ-7, median (IQR)	9	(7–12)	11	(7–16.25)	7	(7–9)	<0.001 **
≥14, n (%)	34	(21.25%)	34	(37.78%)	0	(0%)	<0.001 **
Nine-step test MPD value, median (IQR)	8.5	(4–12.88)	8.25	(3–12.5)	9.5	(4.88–14)	0.157

Mann–Whitney U test. Chi-square test. Fisher’s exact test. ** *p* < 0.05. CRS: Chronic rhinosinusitis ETDQ-7: seven-item Eustachian Tube Dysfunction Questionnaire ETF: Eustachian tube function IQD: interquartile range MPD: maximal peak pressure difference Nine-step test: nine-step inflation–deflation tympanometric Eustachian tube function test.

**Table 3:** Correlations of ETDQ-7 Total Score and Nine-Step Test MPD Value.

	ETDQ-7 Total Score		Nine-Step Test MPD Value	
	R_s_	*p*-Value	R_s_	*p*-Value
Nine-step test MPD value	−0.098	0.217	1.00	
Age	0.32	<0.001	−0.21	0.007

Spearman’s rho. ETDQ-7: seven-item Eustachian Tube Dysfunction Questionnaire Nine-step test: nine-step inflation–deflation tympanometric Eustachian tube function test MPD: maximal peak pressure difference.

## Data Availability

The data presented in this study are available on request from the corresponding author due to patient confidentiality concerns.
